# Parental encouragement of healthy behaviors: adolescent weight status and health-related quality of life

**DOI:** 10.1186/1471-2458-14-369

**Published:** 2014-04-16

**Authors:** Laura Nicholls, Andrew J Lewis, Solveig Petersen, Boyd Swinburn, Marj Moodie, Lynne Millar

**Affiliations:** 1 School of Psychology, Faculty of Health, Deakin University, 221 Burwood Highway, Burwood, Vic 3125, Australia; 2 WHO Collaborating Centre for Obesity Prevention, Faculty of Health, Deakin University, 221 Burwood Highway, Burwood, Vic 3125, Australia; 3 Child and Adolescent Psychiatry, Clinical Sciences, Umeå University, Umeå, Sweden; 4 School of Population Health, University of Auckland, Auckland, New Zealand; 5 Deakin Health Economics, Faculty of Healthy, Deakin University, 221 Burwood Highway, Burwood, Vic 3125, Australia

**Keywords:** Adolescence, Obesity, Wellbeing, Quality of life, Parent-child relations, Social support

## Abstract

**Background:**

Obesity is a major health concern for adolescents, with one in four being overweight or obese in Australia. The purpose of this study was to examine the moderation effect of parental encouragement of healthy behaviors on the relationship between adolescent weight status and Health-Related Quality of Life (HRQoL).

**Methods:**

Baseline data were collected from 3,040 adolescents participating in the *It’s Your Move* project, conducted in the Barwon South-West region of Victoria, in 2005. The Paediatric Quality of Life Inventory was used to measure HRQoL, and parental encouragement was derived from purposely designed self-report items. Weight status was calculated according to World Health Organization growth standards from measured weight and height. Linear regression analyses modeled direct relationships and interaction terms. Analyses were adjusted for age, sex, physical activity level, nutrition and school attended.

**Results:**

Higher levels of parental encouragement, as compared to low encouragement, were positively associated with higher global HRQoL scores, particularly in the physical functioning domain. To a lesser degree, high parental encouragement was also associated with higher scores on the psychosocial domain. Obese weight status showed a significant association with lower HRQoL on all scales. Parental encouragement significantly moderated the inverse relationship between overweight status and physical wellbeing.

**Conclusions:**

Findings suggest that parental encouragement of healthy behavior is associated with increased HRQoL scores for adolescents. Whilst more research is needed to validate the significant interaction effect, main effects suggest that parental encouragement of healthy behavior is an important factor in adolescent wellbeing and should be considered when developing prevention and clinical interventions for obesity.

## Background

Adolescent obesity is a major health concern across most Western societies, including Australia, with one in four Australian adolescents overweight or obese [1]. The World Health Organization (WHO) defines overweight and obesity in children and adolescents (aged 5-19) as having a standardized body mass index (BMI-z) greater than one or two standard deviations (SD) above the mean, respectively [2]. The physical health implications of adolescent obesity are numerous and widely understood, including increased risk of asthma, cardiovascular risk factors and chronic inflammation [3]. However, there is emerging evidence to suggest that physical health conditions like obesity and cardiovascular disease also share a direct relationship with mental health and wellbeing [4,5]. These conditions are complex, and interventions need to be aimed at the individual and the environment in which children live. The home environment is important as including parenting in interventions for adolescents with a range of mental health and psychosocial issues has generally been found to increase the efficacy of interventions, as well as promoting whole of family health and mental health [6]. Therefore, the impact of obesity on psychosocial problems in adolescents is one area of research being explored [7].

Health-Related Quality of Life (HRQoL) is a construct commonly used to conceptualize both physical and psycho-social functioning and wellbeing [8]. Including measures of social, physical and emotional functioning [9], HRQoL is often captured in adolescents with generic tools like the Pediatric Quality of Life Inventory (PedsQL) [10]. A systematic review of 22 studies, which pooled a total sample of 104,093 participants, investigated the effect of weight status on adolescents’ and children’s HRQoL [8]. Twelve studies revealed a significant inverse relationship between weight status and global HRQoL in both community and clinical samples. Pooled analyses of studies utilizing the PedsQL (n = 12,835, k = 13) indicated that weight status was linearly related to both poorer self-reported and parent reported HRQoL. This relationship was verified for global HRQoL and its psychosocial and physical sub domains.

Given the established link between adolescent obesity and poorer HRQoL outcomes, one avenue for further exploration is the role of intervening mechanisms or conditional factors. Findings from a study investigating the inverse relationship between obesity and depression has suggested that the relationship is much more complex than a direct causal chain whereby increases in weight lead to increases in depressive symptoms [11]. The authors proposed that moderation analysis, within a well powered sample, is the next step to fully understanding how the relationship functioned between obesity and depression. Testing moderation effects may uncover the factors which exacerbate or reduce the negative impact of obesity on HRQoL in adolescents.

Previous research investigating the effect of family factors on the inverse relationship between weight status and HRQoL has primarily focused on adolescent perceived social support. Zeller and Modi [12] found that perceived social support from classmates was a strong predictor of quality of life; however, the greatest level of support came from parents and friends. The authors concluded that overall provision of support is an important target for obesity intervention and is related to improved HRQoL outcomes [12]. Whilst an inverse relationship was not supported between weight and quality of life, social support was also found to be an important factor in the wellbeing of a clinically overweight sample of adolescents [13]. Expanding on this research, Herzer, Zellar, Rausch and Modi [14] investigated social support providers and obesity-specific HRQoL in 74 obese adolescent and caregiver dyads, and found that parents and close friends should be included in obesity prevention and treatment interventions, as they were the most valued providers of emotional appraisal and instrumental support for obese youth. Interestingly, only classmates were found to significantly influence HRQoL [14]. Taken together these studies highlight the importance of supportive networks for overweight or obese adolescents in any consideration of their overall functioning.

These studies highlight some common limiting characteristics of the HRQoL and social support literature and suggest areas to be addressed by future research. Firstly, studies utilized small samples, only including adolescents with overweight or obesity [12-14], thus it remains unclear whether social support influences the HRQoL differently in this group than in adolescents with normal weight. A number of studies found that a lack of social support was associated with reductions in the HRQoL of adolescents with obesity [12-14]; however, it remains unclear whether a lack of social support augments the impact of increasing weight on HRQoL, or is an independent risk factor. Another general limitation was that these studies addressed clinical samples seeking treatment for their weight. Treatment-seeking adolescents may suffer greater functional impairment and possess greater motivation for change than adolescents in the community, and therefore may not be representative of overweight adolescents in the population [8]. Also, seeking treatment may already be indicative of being part of a supportive social network, as the family is motivated to take part in a weight loss intervention [12]. Herzer et al. [14] also concluded that receiving general support may not be the best predictor of obesity-specific HRQoL. The authors proposed that support of obesity-specific behaviors such as parental encouragement to engage in healthy eating and physical activity may be an important direction for future research [14].

This study aims to determine whether the relationship between HRQoL and obesity is moderated by parental encouragement of a healthy lifestyle among adolescents. It was predicted that greater encouragement of healthy lifestyle behaviors from parents would be associated with higher HRQoL scores, and that parental encouragement of healthy lifestyle behaviors would moderate an inverse relationship between weight status and HRQoL.

## Methods

### Study design and sample

Data were drawn from the first wave of *“It’s Your Move!”* (IYM); a community-based obesity prevention project, aimed at increasing community capacity to promote healthy eating and physical activity in adolescents. Full details of the design are available in previous publications [15-18]. In brief, the study was located in 12 secondary schools across the Barwon-South West region of Victoria, Australia, which had an estimated population of 350,109 people [17]. Of the 6,013 students invited to take part in baseline data collection, 3,040 students participated (51% response rate). No data were collected on non-respondents but the demographics of the sample were representative of the region. The design of the current study is cross-sectional and derived from baseline data from IYM. All students and their parents gave written consent to their participation in the project. Ethics approval for the IYM project was obtained from the Deakin University Human Research Ethics Committee (EC 37-2004) and it was registered as a trial (ACTRN #12607000257460).

### Measures

#### *The adolescent behaviors, attitudes and knowledge questionnaire (ABAKQ)*

Demographic data were collected via paper questionnaires while Personal Diary Assistants (hand-held mobile devices, Hewlett-Packard iPAC Pocket PC) were used to collect self-reported behavioral and HRQoL data. It took students approximately 15-20 minutes to complete these questionnaires [19].

This purpose designed questionnaire collected self-reported information on physical activity level, nutrition, leisure and sedentary patterns, and perceptions and attitudes about school, body size, family and neighborhood [19]. Most of the food and nutrition behaviour questions were either taken directly from, or adapted from, existing large surveys such as 1995 Australian National Nutrition Survey [20], National Children’s Nutrition Survey which was used in New Zealand in 2002 [21] and 1996 Dietary Key Indicators Study [22]. The survey was initially piloted in 95 students in Australia [19]. Items on daily perceived physical activity and serves of fruit and vegetables were abstracted from this survey as covariates. Perceptions of family items were used to form the parental encouragement factor in the current study.

#### *Health-related quality of life (HRQoL)*

Adolescents self-reported HRQoL via the Paediatric Quality of life inventory (PedsQL) 4.0, generic module for 13-18 year olds. The PedsQL, developed by Dr James W. Varni, has sound psychometric properties [10,23] and is widely used. In the current study we report a global score that totals all 23 items, a psychosocial summary score, which combines the 15 items assessing emotional (“I feel sad”), social (“I have trouble getting along with other teenagers”), and school functioning and wellbeing (“It is hard to pay attention in class”), and a physical score derived from eight items (“It is difficult for me to run”) [24]. After reverse scoring and lineal transformation, scores ranged from 0-100, with a higher score indicating better HRQoL [23,25].

#### *Anthropometry and weight status*

Anthropometric data were collected by trained research staff using standardized protocols [26]. Briefly, students’ height was measured to the nearest 0.1 cm by use of a portable stadiometer (Surgical and Medical PE87) and a TANITA Body Composition Analyzer (Model BC 418, Wedderburn, Australia) was used to measure body weight. Other anthropometric measures included body composition (percentage body fat), BMI (weight in kg/height in m^2^) and BMI-z score, calculated using the World Health Organization (WHO) Reference 2007 [2]. Weight status was determined using BMI z-scores in accordance with WHO standards for children aged 5 to 19 years which states that thinness is equivalent to a BMI z-score <-2SD, normal weight is defined as the range between ≥-2SD and ≤+1SD BMI z-score, overweight is classified as the range between >+1SD and ≤+2SD, and obesity >+2 SD [2].

As less than 2% of participants were classified in the thinness range, normal weight and thinness were combined.

#### *Parental encouragement*

Perceived parental encouragement for physical activity and healthy eating were self-reported by adolescents via four items on the ABAKQ. The questions were scored on a four-point Likert scale (0 = Not at all, 1 = A little, 2 = Some, 3 = A lot) and asked about parental levels of encouragement of healthy behaviors. There was also an option of not living with either a female or male caregiver (where relevant) and those cases (n = 303) were treated as missing and deleted case-wise from analyses. The four parental encouragement questions were as follows: “How much does your mother (or female caregiver) encourage you to eat healthy foods?”; “How much does your father (or male caregiver) encourage you to eat healthy foods?”; “How much does your mother (or female caregiver) encourage you to be physically active or play sports?”; “How much does your father (or male caregiver) encourage you to be physically active or play sports?”.

To investigate whether the parental encouragement items were individual variables, or could be combined to form a meaningful factor, principal factor extraction was performed through Stata version 12 (StataCorp LP, College Station, Texas, USA, 2011). Factors were retained on the basis of Kaiser’s criterion (over 0.6 which is considered adequate for good factor analysis) and one distinct factor was found to underlie adolescent responses to the parental encouragement items [27]. This factor had an eigenvalue greater than one (1.95), and explained 75% of variance. With a cut of 0.32 for inclusion of a variable in interpretation, all four items loaded on the factor [27]. Varimax and promax rotation were used but the factor was not improved so a factor score for a parental encouragement of healthy behaviors factor was generated from the original factor analysis. This factor was then tertiled into low (1), medium (2) and high (3) parental encouragement of healthy behaviors.

### Covariates

Covariates included daily physical activity, serves of vegetables and fruit consumed daily, age in years, sex and the school attended. There is evidence that actual physical activity [28] and consuming a healthy diet independently influence mental health [29]. Therefore these specific covariates were included because the authors wanted to isolate the independent variables of parental encouragement to test if there was an influence above and beyond the behaviors. Full details of measures can be found in previous publications [15-18].

### Data analysis

Descriptive statistics were calculated for the total sample on all variables. Data met the assumptions of normality and there was less than 2% missing. Multiple linear regression analyses were conducted with the independent variables being weight status and parental encouragement and the dependent variables being global HRQoL, psychosocial, and physical functioning and wellbeing. Following the regression analysis, predicted margins were calculated. The unadjusted model (Model 1) included the independent and dependent variables only and the adjusted model (Model 2) included Model 1 plus the covariates: age, sex, school attended, fruit and vegetable serves and amount of daily exercise. Moderation effects were tested using interactions between weight status (normal/thinness, overweight and obesity) and parental encouragement (low, medium and high) with HRQoL. Moderation and main effects were tested with regression models, and significant interactions were graphed to aid interpretation. All analyses were performed in Stata version 12 (StataCorp LP, College Station, Texas, USA, 2011). A probability level was set at *p* < 0.05 to indicate statistical significance.

## Results

### Sample characteristics

Descriptive statistics of the baseline characteristics of participants (n = 3,040) are shown in Table 1. The sample comprised 1,706 (56.12%) males and 1,334 (43.88%) females, the mean age of participants was 14.62 years and the majority of adolescents identified as European Australian (94%). A correlation analysis revealed that global PedsQL scores were very strongly and positively correlated with psychosocial (*r* = 0.96, *p* < .05) and physical scores (*r* = 0.80, *p* < 0.05). Psychosocial and physical PedsQL scores also shared a moderate positive correlation (*r* = 0.62, *p* < .05).

**Table 1:** Participant characteristics

**Characteristic**	**Total**
	**N =3040**
Age in years (Mean ± 1SD)	14.62 ± 1.38
Gender, n (%)	
Boys	1,706 (56.12)
Girls	1,334 (43.88)
Anthropometrics (Mean **±** 1SD)	
Weight in kg	59.61 ± 13.41
Height in meter	164.84 ± 9.68
Body mass index	21.78 ± 3.81
Weight status, n (%)	
Normal/thinness	2,051 (68.41)
Overweight	660 (22.01)
Obesity	287 (9.58)
HRQoL (Mean ± 1SD)	
Global score	78.27 ± 11.42
Psychosocial score	74.30 ± 13.24
Physical score	85.75 ± 11.07
Global HRQoL x	
Weight status, mean (±1SD)	
All	77.86 (10.98)
Normal/thinness	78.98 (10.89)
Overweight	77.73 (11.61)
Obese	74.48 (13.81)
Psychosocial HRQoL x	
Weight status, mean (±1SD)	
All	72.68 (13.54)
Normal/thinness	75.02 (12.69)
Overweight	73.66 (13.534)
Obese	70.68 (15.76)
Pysical HRQoL x	
Weight status, mean (±1SD)	
All	87.58 (10.18)
Normal/thinness	86.40 (10.58)
Overweight	85.35 (11.15)
Obese	81.61 (13.35)
Parental encouragement, mean (±1SD)	
Low	-1.08 (0.53)
Medium	0.12 (0.31)
High	1.00 (0.16)
Parental encouragement x	
Weight status mean (±1SD)	
All	.04 (.79)
Normal/thinness	-.02 (.93)
Overweight	.02 (.94)
Obese	.12 (.92)
Physical activity in min. (Mean ± 1SD)	46.28 ± 29.63
Daily vegetable intake, n (%)	
≤ 1 serve	1,301 (43.40)
> 1 serve	1,697 (56.60)
Daily fruit intake, n (%)	
≤ 1 serve	711 (23.72)
> 1 serve	2,287 (76.28)

*Note. SD* standard deviation, *kg* kilograms, *m* meters, *BMI* body mass index, *BMI-z* standardised BMI defined by the World Health Organization Reference, 2007, Weight Status defined according to WHO cut points (2010), *HRQoL* health-related quality of life, percentages may not equal 100 due to rounding.

### Parental encouragement, weight status and global HRQOL

Linear regression analysis tested the moderating effect of parental encouragement on the relationship between weight status and global HRQoL (Table 2). There were no interactions; however, there were significant main effects. Medium and high levels of parental encouragement (compared to low) were significantly related to higher global HRQoL in the unadjusted model (Model 1). When compared to those who were normal weight, those adolescents who are overweight had similar HRQoL, however those with obesity had significantly lower HRQoL (*p* = .011). All relationships remained significant in the adjusted model (model 2), although the effect was not as strong. The total variance explained by Model 1 was 5% (*R*^2^ = .05, *p* < .001), whereas in model 2, 9% was accounted for (*R*^2^ = .09, p < .001).

**Table 2:** Linear regression analysis summary for variables predicting global health-related quality of life (HRQoL), and psychosocial and physical functioning and wellbeing and interaction effects

		**B**	**SEB**	***p***
Global HRQoL	Low PE (ref)			
	Medium PE	1.76	0.61	.004**
	High PE	3.60	0.64	<.001***
	Normal weight (ref)			
	Overweight	-.41	0.89	.644
	Obesity	-3.32	1.35	.014*
Weight status x PE				
	Overweight x Medium PE	-1.86	1.27	.143
	Overweight x High PE	-1.44	1.25	.251
	Obese x Medium PE	-.33	1.85	.860
	Obese x High PE	-2.49	1.81	.169
Psychosocial	Low PE (ref)			
	Medium PE	1.32	0.72	.068
	High PE	3.49	0.76	<.001***
	Normal weight (ref)			
	Overweight	-0.75	1.05	.473
	Obesity	-3.43	1.60	.032*
Weight status x PE				
	Overweight x Medium PE	-1.39	1.50	.354
	Overweight x High PE	-1.52	1.48	.307
	Obese x Medium PE	.47	2.19	.829
	Obese x High PE	-3.01	2.14	.160
Physical	Low PE (ref)			
	Medium PE	2.62	.58	<.001***
	High PE	3.83	.61	<.001***
	Normal weight (ref)			
	Overweight	.24	.85	.776
	Obesity	-3.11	1.28	.015*
Weight status x PE				
	Overweight x Medium PE	-2.77	1.21	.022*
	Overweight x High PE	-1.32	1.19	.267
	Obese x Medium PE	-1.86	1.76	.292
	Obese x High	-1.56	1.72	.365

*Note. PE* parental encouragement of healthy behaviours; HRQoL measured by the Pediatric Quality of Life Inventory (PedsQL); Weight status defined according to WHO 2007 Reference cut points (2); Model 2: Adjusted regression coefficients, for daily adolescent physical activity (in minutes), serves of vegetables and fruit consumed daily (1 = ≤1, or 2 = ≥ 1 serve), age in years, gender and school attended, for weight status and parental encouragement on global HRQOL, Psychosocial and Physical functioning and wellbeing; *p < .05, **p < .01, ***p < .001.

### Parental encouragement, weight status and psychosocial functioning and wellbeing

The relationship between parental encouragement and weight status and psychosocial functioning and wellbeing (Table 2) were then tested via linear regression analysis. No significant moderation effects were found. Investigation of significant main effects in the unadjusted model (Model 1) revealed that higher levels of parental encouragement were associated with higher psychosocial functioning and wellbeing. When compared to normal weight status, obese weight status was related to poorer psychosocial functioning and wellbeing, but being overweight was not significantly different. After adjusting for covariates (model 2), the results remained for high levels of parental encouragement, but medium levels of parental encouragement were no longer related to higher psychosocial functioning and wellbeing. The unadjusted model explained 3% of the total variance (*R*^2^ = .03, *p* < .001), whereas the adjusted model explained 6% of total variance (*R*^2^ = .06, *p* < .001).

### Parental encouragement, weight status and physical functioning and wellbeing

**Figure 1:**
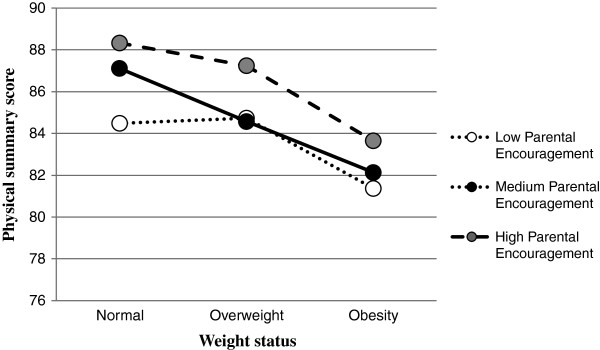
Moderation by levels of parental encouragement on weight status and physical health-related quality of life.

The final linear regression tested the relationship between parental encouragement, weight status and physical functioning and wellbeing (Table 2). The unadjusted model (Model 1) reveals that the relationship between weight status and physical functioning and wellbeing was significantly moderated by parental encouragement (*p* < .05). The significant interaction remained after adjustment for covariates (model 2). Compared to normal weight, those adolescents with overweight that received medium levels of parental encouragement had similar HRQoL as those who received low parental encouragement. To illustrate this effect and to facilitate comparison of results, the interaction between weight status and parental encouragement for physical functioning and wellbeing was plotted for the adjusted model (Figure 1). Investigation of main effects in the unadjusted model revealed that when compared to those who were normal weight, only physical functioning and wellbeing in adolescents with obesity was significantly lower. Compared to low parental encouragement, medium and high levels were significantly related to better physical functioning and wellbeing. All results remained significant in the adjusted model, although the effect was not as strong. Model 1 explained 6% of total variance (*R*^2^ = .06, *p* < .001), whereas model 2 explained 12% of total variance (*R*^2^ = .12, p < .001).

## Discussion and conclusions

Our hypotheses that greater encouragement of healthy lifestyle behaviors from parents would be associated with higher HRQoL scores, and that parental encouragement of healthy lifestyle behaviors would moderate the relationship between weight status and HRQoL, were partly supported by the results of this study. Parental encouragement of healthy behaviors was found to significantly moderate the relationship between weight status and physical functioning and wellbeing in the adjusted model. Two explanations may account for this finding. As was illustrated in Figure 1, receiving a medium or high amount of parental encouragement (compared to low) seemed to be protective of physical functioning and wellbeing among normal weight adolescents. However adolescents in the overweight or obese group had similar physical functioning and wellbeing at low and medium parental encouragement. This may indicate that high levels of parental encouragement are needed to protect the physical functioning and wellbeing in adolescents with overweight or obesity. Since only the physical functioning scale shows this result, it may be indicative of the adolescent’s perceived physical limitations that come with increased body size, however more research is required to elucidate the obesity-related factors that may impact on functional difficulties [8]. However, previous research has found familial social support to be important in improving adolescent HRQoL [12-14], therefore the direction of the interaction is contrary to what would be expected. Further consideration is required to determine whether the interaction was a chance finding in this sample, or has meaningful implications for the development of future interventions.

Adolescents experience many competing influences during this important developmental period including peers and school [30]. Weight status, parental encouragement and the covariates examined by this study explained only a small proportion of the total variance in global HRQoL, including psychosocial and physical functioning and wellbeing. Therefore moderation may not have been supported in this study because the relationship is influenced by other factors. For example, it may be that peer support, rather than parental, is more influential on weight and HRQoL as there is the tendency for a greater conformity to peer groups as children reach adolescence [30]. Low parental encouragement may also be an independent risk factor for HRQoL outcomes in adolescents, regardless of weight status. Compared to low, high parental encouragement to engage in healthy behaviors was related to improvements in global, psychosocial and physical functioning HRQoL scores by almost four points.

The significant main effects of the current study expand upon findings from past research, and taken together can be used to help ensure that future obesity interventions also target quality of life. Zeller and Modi [12] and Herzer et al.[14] both found perceived classmate support to be a significant predictor of HRQoL in a small, treatment-seeking sample of obese adolescents. The current research builds upon this finding by examining perceived parental encouragement in a large, community-based sample. Collectively, it is likely that parent and classmate encouragement play a unique role in fostering positive HRQoL outcomes in adolescents regardless of weight status. The significant positive association between parental encouragement of healthy behaviors and HRQoL is also in line with past research that has investigated the effect of behavior-specific encouragement, from parents and families, on other psychosocial outcomes [31,32].

A major strength of the study is being the first of its kind to examine the moderating role of parental encouragement of health behaviors on the inverse relationship between weight status and HRQoL. Also the use of a large community-based sample and objective measurement of anthropometric outcomes were advantageous. However, obtaining data from a large community-based study has its restraints; the ability to gather in-depth and specific information on all study variables is relinquished and complex constructs are often represented by only a few items. For example, the measure of parental encouragement used in this study can act only as an indicator as it was formed using four survey questions. The extent to which parents were encouraging of healthy behaviours was self-reported by adolescents, which may be subject to recall issues and participants responding according to social desirability. Furthermore, asking adolescents about the level of encouragement they receive from their parents lacks objectivity. Concordance of child and parent perceptions of familial support is approximately 70%, according to a recent study [33]; however, considering that conformity to parental control decreases as children reach adolescence [30], agreement may not be so high between adolescents and their parents. Therefore, collecting data from multiple sources may be needed to verify the findings of this study. Due to the cross-sectional design of the current study relationships among variables should not be interpreted as causal. A major limitation was the response rate of around 50%. Although quite low, this response rate is similar to other similar community-based interventions [34,35]. Lastly, generalizability was limited as the sample was predominately European Australian and the geographic locations from which they were drawn were quite homogenous.

These findings suggest that irrespective of weight status, physical activity level or diet quality, age, sex and school attended, it may be important for parents to be encouraging of healthy eating and physical activity practices. In doing so, parents can help increase global, psychosocial and physical functioning of their adolescent offspring. Furthermore, variation found between HRQoL subscales indicates that some areas of functioning may be more heavily influenced by parental encouragement and weight than others. Specifically, only high levels of parental encouragement were associated with increments in psychosocial functioning, whereas both medium and high levels of encouragement are sufficient to significantly increase the physical and global functioning of adolescents. The results may also have significant implications for developing interventions. Parental encouragement of healthy behaviors has been identified as a factor particularly impacting on physical functioning. Therefore parental encouragement can form an additional target for obesity interventions, especially those involving physical exercise. Rather than focusing on the difficult task of weight reduction [8], targeting parents to increase their levels of encouragement of healthy behaviors, as a mechanism to increase HRQoL, may be an effective strategy for reducing adolescent obesity.

This study represents an initial exploratory step towards establishing whether the inverse relationship between weight and HRQoL differs as a result of parental encouragement of healthy behaviors in adolescents. Future studies should investigate the longitudinal relationship between increasing weight and HRQoL, and the role of moderating factors. This will shed light on the temporal ordering of variables, thus where and when to intervene to prevent or reverse the negative effect of obesity on HRQoL outcomes. Prospective moderation analyses should focus on additional sources of encouragement, including parents, grandparents, classmates, friends and teachers using multi-informant and comprehensive measures.

## Abbreviations

ABAKQ: The adolescent behaviors attitudes and knowledge questionnaire; BMI: Body mass index – weight(k)/height(m^2^); BMI-z: Standardized body mass index (WHO growth standards); HRQoL: Health related quality of life; IYM: *“It’s Your Move!”*; PedsQL: Pediatric quality of life inventory; SD: Standard deviation; WHO: World Health Organization.

## Competing interests

The authors declare that they have no competing interests.

## Authors’ contributions

LN drafted the manuscript, participated in the design of the study and performed the statistical analysis. AL and LM conceived the study, participated in its design and coordination and helped draft the manuscript. LM also performed statistical analysis. MM, BS and SP were involved in revising the manuscript critically for important intellectual content. All authors have read and given final approval of the version published and agree to be accountable for all aspects of the work.

## Pre-publication history

The pre-publication history for this paper can be accessed here:

http://www.biomedcentral.com/1471-2458/14/369/prepub
